# Improving care for SUD patients with complex trauma–relationships between childhood trauma, dissociation, and suicidal behavior in female patients with PTSD and SUD

**DOI:** 10.3389/fpsyt.2022.1047274

**Published:** 2023-01-12

**Authors:** Piotr A. Gidzgier, Melav Bari, Mayte López-Atanes, Annett Lotzin, Johanna Grundmann, Philipp Hiller, Barbara Schneider, Ingo Schäfer

**Affiliations:** ^1^Department of Psychiatry and Psychotherapy, University Medical Center Hamburg-Eppendorf, Hamburg, Germany; ^2^Department of Psychiatry and Psychotherapy, Center for Interdisciplinary Addiction Research, University Medical Center Hamburg-Eppendorf, Hamburg, Germany; ^3^Department of Addictive Disorders and Psychiatry, LVR-Klinik Cologne, Hamburg, Germany

**Keywords:** dual diagnosis, addiction, dissociation, PTSD, suicidal behavior, childhood trauma

## Abstract

**Background:**

Posttraumatic disorders are among the most frequent co-occurring diagnoses in patients with substance use disorders (SUD). Individuals with this dual diagnosis often present with special treatment needs, especially after childhood traumatic experiences (CT). Along with posttraumatic stress disorder (PTSD) and dissociative symptoms, suicidal behaviors belong to the clinical challenges in this group of patients and may influence the course and outcome of SUD treatment. Therefore, a better understanding of the relationships between different forms of CT, psychopathology and suicidal behaviors seems to be important to tailor adequate concepts of care.

**Materials and methods:**

We examined 343 female patients with SUD and Posttraumatic stress disorder (PTSD). All patients completed the Childhood Trauma Questionnaire (CTQ), the Dissociative Experiences Scale-Taxon (DES-T) and the Structured Clinical Interview Axis I Disorders (SCID-I). To determine relationships between different symptoms with potential importance for concepts of treatment, we conducted analyses of moderated mediation for different models. We examined the direct and indirect effects of associations between the type of CT, dissociation and suicidal behavior, as well as the moderation effect of PTSD.

**Results:**

All participants met DSM-criteria for either full PTSD (75.2%) or subsyndromal PTSD (24.8%). Almost all (94.5%) received at least one substance dependence diagnosis and the remaining 5.5% met substance abuse criteria. Most participants (93.3%) reported at least one type of childhood trauma. In all models, dissociation was a risk factor for suicidal ideation (SI) and for suicide attempts (SA). In both, participants with subsyndromal PTSD and participants with full PTSD, dissociation mediated the relationship between childhood sexual abuse and SI as well as SA. Moreover, we report direct effects between different childhood traumas and SI and SA. Furthermore, emotional abuse was a significant predictor of dissociation.

**Discussion:**

In our sample of female patients with SUD and co-occurring PTSD, dissociation significantly increased suicidal behavior and served as a mediator of the relationship between childhood sexual abuse and suicidal behavior. Our findings underline the need to include interventions to address dissociative symptoms and other more complex consequences of childhood trauma into concepts of care for patients with SUD.

## 1. Introduction

Posttraumatic disorders are among the most frequent co-occurring diagnoses in patients with substance use disorders (SUD) (1). Patients with this comorbidity often report experiences of repeated childhood sexual and physical abuse and present complex treatment needs (2). While systematic studies on this topic are lacking so far, it can be assumed that a significant proportion of patients with SUD and comorbid PTSD fulfills the diagnosis of complex PTSD (cPTSD) (3). Although the concept of cPTSD was proposed about 30 years ago (4), it had not been adopted as a formal diagnosis before the 11th revision of the World Health Organization's International Classification of Diseases (ICD-11) (3). In addition to the core symptoms of PTSD, the diagnosis of complex PTSD includes three additional groups of symptoms: emotion regulation difficulties, difficulties maintaining relationships and negative self-concept (4). These symptoms may disrupt engagement in treatment, reduce the capability to attain new skills and knowledge and disturb resistance of the urge to use substances (5). For instance, in a randomized trial of contingency management compared to standard treatment in 146 cocaine- or heroin-dependent outpatients, complex PTSD symptoms were related to poorer treatment outcomes independent of the effects of demographics, baseline substance use, and treatment modality (6). The Diagnostic and Statistical Manual of Mental Disorders (DSM-5) (7) takes a different approach to describe more complex representations of PTSD. In addition to the inclusion of changes of cognitions and mood in the symptoms of PTSD, it allows to diagnose a dissociative subtype of PTSD (D-PTSD). D-PTSD is characterized by at least one of two characteristic features: dissociative depersonalization and derealization (7).

Along with the symptoms of PTSD and dissociation, suicidal behavior belongs to the most prominent clinical problems in patients concerned. For instance, in a study among veterans, SUD comorbidity with PTSD greatly elevated suicidal ideation risk (8). Moreover, in a study among 459 patients with SUD, patients with D-PTSD reported significantly more current suicidal ideation (SI) and more suicide attempts (SA) as compared to patients with PTSD without dissociative features (9). Despite their clinical importance, relationships between the symptoms of (complex) PTSD and suicidal behavior in SUD patients remain unclear. One hypothesis is that, in addition to the symptoms of PTSD, third factors, namely childhood trauma (CT), increase the risk for suicidal behavior later in life (10–12). Studies among patients in treatment for substance abuse found that CT was associated with a very high probability of SA. Over 75% of men and 87% of women which reported CT also had a history of SA (13, 14). A study of women in residential treatment for drug and alcohol abuse reported that childhood sexual abuse was uniquely associated with SA (15). Finally, a number of studies showed that dissociative symptoms might be a strong mediator between childhood trauma and SA (16–18).

Relationships between different types of CT, trauma-related symptoms and suicidal behavior may have implications for treatment concepts. If, for instance, dissociation would be a determinant of suicidal behavior, the integration of interventions to reduce dissociative symptoms into SUD treatment would be of special importance. This could be seen as less critical if both symptom areas would be independently related to childhood trauma. Similarly, specific interventions for victims of different types of childhood trauma, i.e., sexual abuse vs. emotional abuse or different forms of neglect, for SUD patients with traumatic experiences would be of higher importance if they would be independently related to suicidal behavior. The aim of this exploratory study therefore was to examine whether dissociation is a mediator between different types of CT and suicidal ideation as well as suicide attempts in female patients with SUD and PTSD. For both types of suicidal behaviors, we examined if the potential relationships were moderated by PTSD status (i.e., full or subsyndromal PTSD).

## 2. Materials and methods

### 2.1. Participants

The present study used data of a randomized controlled trial of a cognitive behavioral treatment for women diagnosed with PTSD and SUD (19). Data was gathered at the University Medical Center Hamburg-Eppendorf, Germany, and four other German research institutions (Bielefeld, Essen, Hannover, Cologne). All study centers were substance abuse treatment departments of the respective hospitals. All study procedures were approved by the ethics committees of the responsible chambers of physicians at each study site (reference number of the leading site: PV4178). Moreover, the trial was registered at the German Clinical Trials Register under the ID DRKS00004288. Study participants were recruited *via* local substance abuse and trauma counseling agencies, psychosocial services, substance abuse and mental health clinics, psychotherapists in private practice and from the community (e.g., adverts in city transport, in magazines, at stores and in online adverts). As the prevalence of PTSD is considerably higher in women with SUD as compared to men (2), the study concentrated on females with this comorbidity. Inclusion criteria were female sex, age 18–65, subthreshold PTSD (i.e., criterion A, B, and either C or D) or full PTSD and a substance use disorder with last substance use within the previous 12 months, both according to DSM-IV criteria (7). Exclusion criteria were current psychosis, severe cognitive impairment and intravenous drug use in the month before study participation. Out of 610 individuals assessed for eligibility, *n* = 234 had to be excluded because they were not eligible (*n* = 123), declined to participate (*n* = 47), were lost to baseline assessment (*n* = 34), were lost for other reason (e.g., inpatient treatment, incomplete screening, imprisonment; *n* = 30) and discontinued baseline assessment (*n* = 33) resulting in a final sample of *n* = 343 participants.

### 2.2. Measures

#### 2.2.1. Childhood trauma questionnaire

The Childhood Trauma Questionnaire (CTQ) (20) collects information on the type and severity of early traumatic experiences. The CTQ is a 28-item self-report questionnaire that assesses physical and sexual abuse, emotional neglect, emotional abuse and physical neglect. Items are rated on a Likert-scale from 1 (never true) to 5 (very often true). The five subscale scores range from 5 to 25. For each of the five subscales, the severity of abuse or neglect can be classified according to defined cutoff scores (none or minimal, low to moderate, moderate to severe, and severe to extreme). A German version of the CTQ demonstrated good internal consistencies, factorial, convergent and discriminant validity (21), also in clinical samples with diagnosed SUD, and PTSD (22). The reliability estimates of CTQ subscales in our study were Cronbach's α = 0.86 for emotional abuse, α = 0.89 for physical abuse, α = 0.96 for sexual abuse, α = 0.88 for emotional neglect, and α = 0.71 for physical neglect.

#### 2.2.2. Dissociative experiences scale-taxon

A subset of eight items of the Dissociative Experiences Scale (23), the so-called DES-Taxon (DES-T), has been proven to be a sensitive self-rating tool to identify pathological dissociation (24). The questions of the DES-T are answered by estimating the percentage of time (ranging from 0 to 100%), in which the subject goes through the experience described (e.g., “Some people sometimes have the experience of feeling that their body does not belong to them”). The mean score is calculated by dividing the total percentage of time by the number of answered items. Reliability testing of the DES-T showed that the scale had good test-retest and good split-half reliability. Internal consistency and construct validity were also described as good (23), also in clinical samples diagnosed with SUD and PTSD (22). The reliability estimate of DES-T scale in our study was Cronbach's α = 0.80.

#### 2.2.3. The structured clinical interview for DSM-IV axis I disorders

The diagnoses of PTSD and SUD were confirmed by using the Structured Clinical Interview for DSM-IV Axis I Disorders (SCID-I) (25). The SCID-I was also used to assess suicidal behavior such as SI and SA. Studies on the SCID-I have shown a good to very good validity and reliability of the instrument (26).

### 2.3. Data analysis

To investigate relationships between childhood trauma, dissociation and suicidal behavior, we 1) examined globally—in both, the PTSD and the subsyndromal PTSD group, the relationships between different types of CT and dissociation with a possible moderation effect of PTSD status (full PTSD and subsyndromal PTSD, respectively), we 2) examined globally—in both, the PTSD and the subsyndromal PTSD group, relationships between different types of CT and suicidal behavior with a possible moderation effect of PTSD status, we 3) examined relationships between dissociation and suicidal behavior, and 4) we examined direct effects of childhood trauma on suicidal behavior as well as indirect effects, taking the influence of dissociation on this relationship into account, as well as the influence of PTSD status on these effects. Both types of suicidal behaviors (suicidal ideation and suicide attempts) were considered separately, i.e., these analyses were performed for each of the two types.

In the first step, the normality of the data distribution was tested in the groups (full PTSD vs. subsyndromal PTSD) that were treated as a moderator of the mediation of the tested variables. The aim was to verify the assumption of normal distribution for continuous variables tested in moderated mediation models. The obtained results indicated that the normality of the distribution could be assumed for the vast majority of variables due to the skewness criterion. The only indicator that slightly exceeded this assumption was dissociation in the group of women with subsyndromal PTSD (*Sk* = 2.19). However, it was found that the direction of the skewness of the subsyndromal PTSD group was identical to skewness of the group of women with full PTSD, which suggests that both groups can be compared. We checked CT and PTSD for multicollinearity. They correlated at < 0.20, meaning that they mutually explained <5% of the variance of each other, and the VIF coefficients between all predictors equalled <5 which means that multicollinearity did not occur. The moderated mediation analysis in Hayes method yields b-values, which is the non-standardized regression coefficient. The Hayes method concentrates on determining the direction of the relationship rather than on measuring its strength. Consequently, we decided to perform a moderated mediation analysis based on the quantitative indicators. The moderated mediation analysis in Hayes method yields *b*-values, which is the non-standardized regression coefficient. The Hayes method concentrates on determining the direction of the relationship rather than on measuring its strength. The level of significance in our study was assumed to be α = 0.05. All analyses were carried out by using the IBM SPSS Statistics 27 (27) software together with the PROCESS 3.5 macro (28).

## 3. Results

### 3.1. Sample characteristics

In total, *n* = 343 treatment-seeking women with SUD and (at least subsyndromal) PTSD were included in the study. On average, the participants were 40.9 years old (*SD* = 11.4; range = 18–65). Completed years of education ranged from 7 to 13 years, with a median of 10 years. Almost all women were born in Germany (*n* = 310, 90.4%). The majority of women were unmarried (*n* = 287, 83.7%) and unemployed (*n* = 267, 77.8%). About half of them had a monthly income of less than €1000 (*n* = 186, 54.2%).

Nine in 10 women (*n* = 324, 94.5%) were diagnosed with a substance dependence, the remaining women (*n* = 19, 5.5%) were diagnosed with substance abuse. Multidrug use was the rule rather than the exception. Eight in 10 women (*n* = 290, 84.5%) were diagnosed with an alcohol use disorder. About half of the women (*n* = 165, 48.5%) fulfilled the diagnostic criteria for a cannabis use disorder. About three in 10 women fulfilled the criteria for a sedative use disorder (*n* = 106, 31.2%), a cocaine use disorder (*n* = 97, 28.5%) and a stimulant use disorder other than cocaine (*n* = 96, 28.2%), respectively; finally, two in 10 women (*n* = 73, 21.3%) were diagnosed with an opiate use disorder. Almost eight in 10 women (*n* = 270, 78.7%) had consumed substances within the last 30 days and six in 10 women (*n* = 226, 65.9%) had previously participated in substance abuse treatment.

The majority (*n* = 258, 75.2%) met the criteria of a full PTSD diagnosis and the remaining individuals (*n* = 85, 24.8%) fulfilled the criteria for subsyndromal PTSD. About one in four women had participated in prior trauma-related treatment (*n* = 80, 23.3%). Nearly half of the women were diagnosed with Major Depression (*n* = 153, 44.6%), and two thirds of the women were diagnosed with an anxiety disorder (*n* = 221, 64.4%). Almost six in 10 women had attempted suicide in their life (*n* = 197, 57.4%) and the same amount (*n* = 197, 57.4%) reported suicidal ideation. Four in 10 women (*n* = 130, 37.9%) reported both suicidal ideation and consecutive suicide attempts. Among the women who attempted suicide the average count of SA was 2.0 (*SD* = 4.61). There was 1 woman who had attempted suicide 71 times and 50 women who had attempted suicide only once.

As defined by our inclusion criteria, all women were exposed to a traumatic event according to DSM-IV. The majority of women (*n* = 320, 93.3%) reported at least one type of childhood abuse or neglect. Eight in 10 women reported at least “moderate to severe” levels of emotional abuse (*n* = 267, 77.8%) or emotional neglect (*n* = 261, 76.1%); seven in 10 (*n* = 249, 72.6%) reported at least “moderate to severe” sexual abuse, six in 10 (*n* = 209, 60.1%) reported at least “moderate to severe” physical neglect; and half of women (*n* = 179, 52.2%) reported at least “moderate to severe” physical abuse.

### 3.2. Relationships between childhood trauma, dissociation, and suicidal ideation

No statistically significant relationships between the different types of CT and dissociation were found on the global level (in both, the PTSD and the subsyndromal PTSD group), and there was no moderation effect of PTSD (both full and subsyndromal) on these relationships (Table 1). The PTSD group was not related to the strength and direction of the presented relationships between CT and dissociation, as well as between CT and SI. This suggests that the obtained relationships for patients were similar in strength and direction regardless of PTSD group. We also didn't find any significant relationships when analyzing the relationships between different types of CT and SI on the global level. However, subgroup analyses showed that there was a positive and significant effect in women with subsyndromal PTSD [*b* = 0.082 (0.010; 0.155); *p* = 0.027], but not with full PTSD [*b* = 0.011 (−0.027; 0.048); *p* = 0.580]. This effect may suggest that a direct link between sexual abuse and SI is more common among women with subsyndromal PTSD rather than with full PTSD. The only variable on the global level that was associated with a significant increase in the frequency of SI in all CT models was dissociation (Table 2).

**Table 1:** Demographic and clinical sample characteristics.

**Characteristics**	**Full PTSD^b^** **(*n* = 258)**	**Subsyndromal PTSD^b^** **(*n* = 85)**	**Total** **(*n* = 343)**
		***n (%)*** ^a^	
Age, mean (SD)	40.03 (11.41)	43.69 (10.83)	40.9 (11.4)
**Marital status**			
Single	153 (59.3)	32 (37.6)	185 (53.9)
Married	36 (14.0)	20 (23.5)	56 (16.3)
Divorced	65 (25.2)	33 (38.8)	98 (28.6)
Widowed	4 (1.6)	0 (0.0)	4 (1.2)
**Employment**			
Unemployed	182 (70.5)	48 (56.5)	230 (67.1)
Minor employment	27 (10.5)	10 (11.8)	37 (10.8)
Part-time employment	23 (8.9)	16 (18.8)	39 (11.4)
Full-time employment	25 (9.7)	11 (12.9)	36 (10.5)
**Children**			
Yes	126 (48.8)	50 (58.8)	176 (51.3)
No	132 (51.2)	35 (41.2)	167 (48.7)
**Substance use disorder** ^b^			
Alcohol	214 (82.9)	76 (89.4)	290 (84.5)
Sedatives	78 (30.2)	28 (32.9)	106 (31.2)
Cannabis	127 (49.2)	38 (44.7)	165 (48.5)
Stimulants	81 (31.4)	15 (17.6)	96 (28.2)
Opiates	55 (21.3)	18 (21.2)	73 (21.3)
Cocaine	73 (28.3)	24 (28.2)	97 (28.5)
**Childhood trauma** ^c^			
Emotional abuse	205 (79.5)	62 (72.9)	267 (78.5)
Physical abuse	139 (53.5)	41 (48.2)	179 (52.2)
Sexual abuse	193 (74.8)	56 (65.9)	249 (72.8)
Emotional neglect	204 (79.1)	57 (67.1)	261 (76.5)
Physical neglect	164 (63.6)	45 (52.9)	209 (61.1)
Dissociation^d^, mean (SD)	11.65 (12.6)	8.5 (12.5)	10.9 (12.6)
**Suicidal behavior** ^d^			
Suicidal attempt	153 (59.3)	45 (52.9)	198 (57.7)
Suicidal ideation	162 (62.8)	36 (42.4)	198 (57.7)
Suicidal attempts count, mean (SD)	3 (4.7)	2.8 (4.2)	2.9 (4.6)

a If not otherwise specified.

b SCID-I.

c CTQ (At least “moderate to severe”).

d DES-Taxon.

**Table 2:** Analysis of moderated mediation in the relationship between early childhood trauma and suicidal ideation.

**Model**	**PTSD**	**CT → Dissoc**.	**Dissoc. → SI**	**CT → SI**	**PTSD**	**Moderated mediation**		**χ^2^**	***p***	***R^2^*** **Cox-Snell**
	↓				↓					
	**CT**→**Dissoc**.				**CT**→**SI**	**LLCI**	**ULCI**			
Emot. Abuse	0.088	−0.165	0.033^**^	0.083	0.012	−0.017	0.023	29,109	< 0.001	0.082
Phys. Abuse	0.129	0.272	0.035^***^	0.154	0.049	−0.031	0.015	25,129	< 0.001	0.071
Sex. Abuse	0.228	0.198	0.032^**^	0.225	−0.071	−0.007	0.024	28,423	< 0.001	0.080
Emot. Negl.	0.017	0.101	0.035^***^	0.068	−0.001	−0.026	0.024	31,617	< 0.001	0.089
Phys. Negl.	0.642	1.913	0.034^**^	0.012	0.019	−0.057	0.004	25,438	< 0.001	0.072

CT, five individual models: emotional abuse, physical abuse, sexual abuse, emotional neglect, physical neglect; Dissoc, dissociation; PTSD, full vs. subsyndromal PTSD; SI, suicidal ideation;

** *p* < 0.01;

*** *p* < 0.001; LLCI, lower limit of the confidence interval of the moderated mediation effect; ULCI, upper limit of the confidence interval of the moderated mediation effect.

Finally, when analyzing direct and indirect effects, we found a direct effect between emotional abuse and dissociative symptoms that occurred only among individuals with full PTSD [*b* = 0.047 (0.001; 0.095); *p* = 0.044]. This could indicate that emotional abuse is more strongly associated with dissociation in patients diagnosed with PTSD than with subsyndromal PTSD. Moreover, it was noticed that there was a direct effect between emotional neglect and SI among women with full PTSD [*b* = 0.065 (0.016; 0.114); *p* = 0.010]. In addition, in women with full PTSD, we found an indirect mediation effect of dissociative symptoms related to sexual abuse and SI [*b* = 0.016 (0.005; 0.032)] which suggests that dissociation might mediate the relationship between sexual abuse and SI.

### 3.3. Relationships between childhood trauma, dissociation, and suicide attempts

The results showed that there were no statistically significant relationships between the examined types of CT and dissociation on the global level and there was no significant moderation effect of PTSD (full vs. subsyndromal) in these relationships (Table 2). Again, PTSD group was not related to the strength and direction of the presented relationships between CT and dissociation as well as between CT and SA, indicating that the obtained relationships for patients were similar in strength and direction regardless of PTSD type. When analyzing the relationships between different types of CT and SA, we found a statistically significant relationship on a global level between physical abuse and SA (*p* = 0.047). This effect may suggest that a direct link between physical abuse and SA is common in both subsyndromal PTSD and full PTSD individuals. We again found that dissociation was a significant variable that was associated with an increase in the frequency of SA in all presented models on the global level (Table 3).

**Table 3:** Analysis of moderated mediation in the relationship between early childhood traumsa and any suicide attempt.

**Model**	**PTSD**	**CT → Dissoc**.	**Dissoc. → SA**	**CT → SA**	**PTSD**	**Moderated mediation**		**χ^2^**	***p***	***R^2^*** **Cox-Snell**
	↓				↓					
	**CT**→**Dissoc**.				**CT**→**SA**	**LLCI**	**ULCI**			
Emot. Abuse	0.088	−0.165	0.027^**^	0.156	−0.034	−0.013	0.019	9,729	0.001	0.056
Phys. Abuse	−0.129	0.272	0.029^**^	0.367^*^	−0.109	−0.025	0.011	2,261	0.001	0.063
Sex. Abuse	0.228	−0.198	0.025^**^	0.118	−0.035	−0.004	0.021	2,602	0.013	0.036
Emot. Negl.	0.017	−0.101	0.028^**^	0.207	−0.052	−0.020	0.020	8,986	0.001	0.054
Phys. Negl.	−0.642	1.913	0.026^**^	0.322	−0.077	−0.049	0.003	7,041	0.001	0.076

CT, Variable differing in the five individual models: emotional abuse, physical abuse, sexual abuse, emotional neglect, physical neglect; Dissoc, dissociation; PTSD, agnosed full-blown or subsyndromal posttraumatic stress disorder; SA, suicide attempt;

* *p* < 0.05;

** *p* < 0.01; LLCI, lower limit of the confidence interval of the moderated mediation effect; ULCI, upper limit of the confidence interval of the moderated mediation effect.

Finally, the analysis of the direct effects for both PTSD groups (subsyndromal PTSD and full PTSD) showed a positive relationship between emotional abuse and SA, both in the subsyndromal PTSD group [*b* = 0.090 (0.004; 0.175); *p* = 0.039] and the full PTSD group [*b* = 0.056 (0.010; 0.102); *p* = 0.018]. This means that emotional abuse is potentially associated with dissociation in patients diagnosed with PTSD and with subsyndromal PTSD. There was also a significant positive direct effect between emotional neglect and SA in the subsyndromal PTSD group [*b* = 0.103 (0.013; 0.194); *p* = 0.025] and the full PTSD group [*b* = 0.051 (0.003; 0.100); *p* = 0.036]. Therefore, the results suggest that emotional neglect can be also associated with dissociation in patients diagnosed with PTSD and with subsyndromal PTSD. In addition, direct effect for the relationship of physical abuse on SA was found only in the subsyndromal PTSD group [*b* = 0.149 (0.045; 0.252); *p* = 0.005)] suggesting the respective association. Moreover, the analysis of the indirect effects showed that in women with full PTSD there was a significant indirect mediation effect of dissociation between sexual abuse and SA [*b* = 0.012 (0.003; 0026)] which suggests that dissociation might mediate the relationship between sexual abuse and SA.

## 4. Discussion

To our knowledge, this is the first study that examined the mediating role of dissociation between different forms of childhood trauma and suicidal behaviors in patients with SUD and PTSD. To gain a better understanding of such associations, we studied a large sample of women with this comorbidity. A sequence of moderated mediation analyses revealed that, in women with full PTSD, dissociation mediated the relationship between childhood sexual abuse and SI, as well as SA. Moreover, our findings suggest that dissociation could be an independent risk factor that increases the frequency of both SI and SA in all models. In addition, our results indicate that in women diagnosed with full PTSD, emotional abuse and emotional neglect might independently increase the risk of both forms of suicidal behaviors, while in women diagnosed with subsyndromal PTSD, emotional abuse, emotional neglect, and physical abuse might only increase the risk of SA. Furthermore, our results seem to imply that emotional abuse may be a predictor of dissociation. The latter is in line with research across different populations (29–31) and has also been reported in previous studies of patients with SUD and PTSD (18, 22).

Our findings underline the importance of dissociative symptoms for suicidal behaviors, especially in survivors of sexual abuse. While other studies reported direct associations between sexual abuse and suicidal behaviors (32, 33), our findings are consistent with the literature that reports a mediating role of dissociative symptoms (34). The fact that these associations were only observed in women with full PTSD suggests that overall severity of psychopathology plays a role and dissociation could be part of cPTSD in these patients. Another interesting aspect concerns the direct links between emotional trauma and suicidal behaviors, which support the results of previous research (35). Again, these relationships were more prominent in patients with full PTSD, where relationships with both SI and SA were observed. In patients with partial PTSD, direct relationships were no longer observed in relation to SI, but still in relation to SA. While our analyses do not allow to answer this question, it could be assumed that patients with full PTSD had been exposed to more complex childhood trauma, explaining the more consistent relationships with suicidal behaviors. For instance, a meta-analysis by Angelakis et al. (36) suggested that all different types of childhood maltreatment were associated with two- to three-fold increased risk for suicide attempts. Complex childhood abuse, however, was associated with a more than five-fold increased risk for suicide attempts in adulthood. Similar results were found for the association between childhood maltreatment and suicidal ideation (36). The direct relationships between emotional trauma and both SI as well as SA suggest other mediators than dissociative symptoms between these forms of childhood trauma and suicidal behaviors. Relevant factors could be low self-esteem as a result of enduring emotional abuse and neglect, which has been reported to mediate the relationship between childhood maltreatment and suicidal ideation (37), but also other consequences of emotional trauma like self-hatred (38) and hopelessness (39).

Our study has implications for the treatment of patients with SUD and PTSD. The findings support claims by previous studies in the fields of PTSD or SUD to specifically address dissociative symptoms, and suggest that this should also be the case in patients with comorbid SUD and PTSD. For instance, research in the past years has indicated that PTSD patients with dissociative symptoms show a poor response to standard trauma therapies and exhibit high levels of attrition from treatment. In their systematic review of dissociation in PTSD, Atchley and Badford (40) therefore conclude, that special interventions to address dissociative symptoms need to be integrated in PTSD treatment and that dissociation should be assessed as a separate outcome. The same has been found for patients with SUD. In a study by Tamar-Gurol et al. (17), 55% of drug dependent patients presenting dissociative symptoms prematurely dropped out of treatment for drug abuse compared to 29% of those without dissociative symptoms. Similarly, Somer (41) found that dissociation predicted lower rates of abstinence among heroin users in treatment and stressed the necessity of addressing trauma-related dissociation to improve their outcomes. While not systematically addressed in treatment for PTSD or SUD so far, evidence for the effectiveness of interventions for dissociative symptoms and dissociative disorders is accumulating. Brand et al. (42) concluded in her review of the dissociative disorders literature that if treatments are explicitly shaped to address complex trauma and dissociation, even highly effected patients may benefit. However, there still is a glaring lack of evidence-based interventions to address dissociation in patients with SUD, one obvious reason for this being that SUD is often an exclusion criterion in studies investigating programs for the treatment of child abuse-related PTSD (40, 43). This lack of interventions is increasingly perceived in the SUD field. For instance, Patel et al. (44), who recently investigated the mediating role of dissociative symptoms between the severity of PTSD and alcohol related problems, stressed the need to develop corresponding treatments. These could, again, come from the trauma field, which has seen strong developments in evidence-based interventions for patients with complex symptoms in recent years. For instance, Skills Training in Affective and Interpersonal Regulation-Narrative Therapy (STAIR-NT) is an evidence-based psychotherapy designed to treat individuals affected by cPTSD (45). It has been shown to be an effective intervention for a variety of populations, including adults and adolescents, males and females, as well as inpatients and community members. An RCT comparing STAIR to treatment as usual (TAU) in VA primary care found significant reductions in PTSD, depression, emotion regulation and social functioning (46). A comparative study of STAIR group vs. TAU among individuals with PTSD and chronic mental illness suggested that it can also be successfully used in groups with special needs (47). An easy to integrate intervention with a direct focus on dissociative symptoms could also be the third phase of the DBT-PTSD program, which focuses on skills training and cognitive strategies to improve emotion regulation and dealing with dissociation (48).

Strengths of our study concern the large clinical sample of females with a dual diagnosis of PTSD and SUD, and the inclusion of a wide range of potentially relevant variables in our analyses. It extends previous research (32, 33) by including PTSD status in the analyses of potential relationships between dissociative symptoms and suicidal behaviors, to examine the impact of other trauma-related psychopathology. A limitation is the use of self-report scales like CTQ and DES-T, while all data regarding suicidality and PTSD were collected by means of clinical interviews. Also, we only included women because of the higher prevalence of co-occurring PTSD in female patients with SUD. Moreover, some characteristics of our sample suggest that it might also not be fully representative of female patients with SUD and PTSD. Indicators for this could be the comparatively high level of education and the low level of women with a migration background. Conclusions about other samples of patients with SUD and PTSD, e.g., male patients and patients with PTSD related to adulthood trauma, must therefore be drawn with caution. Bertule et al. (33), in their study on depression as a mediator between dissociation and SI, found more SI in men than in women. This further highlights the importance to address predictors of suicidal behaviors in male populations of patients with SUD and PTSD in future studies. One limitation of our study is that the moderated mediation analyses in Hayes method yield a non-standardized regression coefficient (*b*-value). Although the method cannot determine the strength of the relationship, it determines the significance and the direction of the relationship—positive vs. negative. The higher *b*-value is only a potential indicator of the effect's strength. It should be mentioned that we did not include substance use in our analyses, which can serve as a mechanism of emotion regulation and interfere with the examined variables in our models. In addition, variables like psychiatric family history might have been of help to further stratify the sample and the inclusion of treatment variables, for instance, current pharmaco- and psychotherapy, might have led to differing results. Future studies should include further potentially relevant variables like emotion dysregulation, depressive symptoms or the presence of a diagnosis of borderline disorder.

In conclusion, our findings suggest that dissociation has direct effects on both suicidal ideation and suicide attempts in patients with the dual diagnosis of SUD and PTSD, and that it mediates the relationship of some forms of childhood trauma, namely childhood sexual abuse, with suicidal behaviors. The direct relationships between emotional trauma and suicidal behaviors suggest that further important mediators, like self-esteem and self-concept, should be addressed in future studies. After several decades of research into co-occurring PTSD in patients with SUD, our findings underline the need to widen the established perspectives and to include interventions for more complex consequences of childhood trauma into concepts of care for patients with SUD.

## Data availability statement

The raw data supporting the conclusions of this article will be made available by the authors, without undue reservation.

## Ethics statement

All study procedures were approved by the Ethics Committees of the responsible chambers of physicians at each study site. The patients/participants provided their written informed consent to participate in this study.

## Author contributions

PG and IS contributed to the conception and design of the study. AL, JG, and PH organized the database. PG performed the statistical analysis and wrote the first draft and final sections of the manuscript. ML-A and MB performed manuscript reviews. All authors contributed to manuscript revision, read, and approved the submitted version.
